# Correlation between MMPs and their inhibitors in breast cancer tumor tissue specimens and in cell lines with different metastatic potential

**DOI:** 10.1186/1471-2407-9-20

**Published:** 2009-01-14

**Authors:** Rita CS Figueira, Luciana R Gomes, João S Neto, Fabricio C Silva, Ismael DCG Silva, Mari C Sogayar

**Affiliations:** 1 Department of Biochemistry, Chemistry Institute, University of São Paulo, São Paulo, SP, Brazil; 2 Hospital do Câncer Alfredo Abrão, Campo Grande, MS, Brazil; 3 Department of Gynecology, Federal University of São Paulo, São Paulo, SP, Brazil

## Abstract

**Background:**

The metastatic disease rather than the primary tumor itself is responsible for death in most solid tumors, including breast cancer. The role of matrix metalloproteinases (MMPs), tissue inhibitors of MMPs (TIMPs) and Reversion-inducing cysteine-rich protein with Kazal motifs (RECK) in the metastatic process has previously been established. However, in all published studies only a limited number of MMPs/MMP inhibitors was analyzed in a limited number of cell lines. Here, we propose a more comprehensive approach by analyzing the expression levels of several MMPs (MMP-2, MMP-9 and MMP-14) and MMP inhibitors (TIMP-1, TIMP-2 and RECK) in different models (five human breast cancer cell lines, 72 primary breast tumors and 30 adjacent normal tissues).

**Methods:**

We analyzed the expression levels of MMP-2, MMP-9 and MMP-14 and their inhibitors (TIMP-1, TIMP-2 and RECK) by quantitative RT-PCR (qRT-PCR) in five human breast cancer cell lines presenting increased invasiveness and metastatic potential, 72 primary breast tumors and 30 adjacent normal tissues. Moreover, the role of cell-extracellular matrix elements interactions in the regulation of expression and activity of MMPs and their inhibitors was analyzed by culturing these cell lines on plastic or on artificial ECM (Matrigel).

**Results:**

The results demonstrated that MMPs mRNA expression levels displayed a positive and statistically significant correlation with the transcriptional expression levels of their inhibitors both in the cell line models and in the tumor tissue samples. Furthermore, the expression of all MMP inhibitors was modulated by cell-Matrigel contact only in highly invasive and metastatic cell lines. The enzyme/inhibitor balance at the transcriptional level significantly favors the enzyme which is more evident in tumor than in adjacent non-tumor tissue samples.

**Conclusion:**

Our results suggest that the expression of MMPs and their inhibitors, at least at the transcriptional level, might be regulated by common factors and signaling pathways. Therefore, the multi-factorial analysis of these molecules could provide new and independent prognostic information contributing to the determination of more adequate therapy strategies for each patient.

## Background

Among diverse cancer types, breast carcinoma stands out for its increasing incidence rates and high mortality worldwide [1]. Like most solid tumors, metastatic disease rather than the primary tumor itself is responsible for death [2-4]. The metastatic process involves a complex cascade of events, including the organized breakdown of the extracellular matrix (ECM) by matrix metalloproteinases (MMPs) [5,6]. Together, the MMPs are able to process or degrade all ECM components. Each ECM element is cleaved by a specific MMP or MMP group [7]. The activity of these proteases is tightly regulated by specific inhibitors, known as tissue inhibitors of MMPs (TIMPs) [7,8]. Consistent with their role in tumor progression, high levels of a number of MMPs have been shown to correlate with poor prognosis in human cancers [9-11]. Surprisingly, high levels of TIMP-1 and TIMP-2 have also been shown to predict adverse prognosis and correlate with tumor aggressiveness in several different human cancers, including breast cancer [12-14]. TIMPs expression profile could be the result of its action as a multifunctional molecule [8].

The RECK metastasis suppressor gene was isolated by screening a fibroblast expression library for cDNAs that induced flat revertants in ν-Ki-ras-transformed NIH3T3 cells [15]. RECK encodes a membrane-associated MMP regulator protein that is able to suppress tumor invasion and metastasis by negatively regulating MMPs involved in carcinogenesis, namely: MMP-2, MMP-9 and MMP-14 (MT1-MMP) [16,17]. Due to these functions, RECK has been described as a good prognosis marker in several tumor types, including breast carcinomas [18,19].

The ECM degradation and, consequently, the invasive and metastatic potential of tumor cells is the result of the umbalance between the activities of these multiple factors that compose the proteases/inhibitors equilibrium [20]. Furthermore, each one of these molecules is involved in different stages and processes during tumor progression [20,21]. Although the expression and activity profile of MMPs, TIMPs and RECK have already been described in several cell line models [22-24], few reports analyze more than one MMP or MMP inhibitor in different cell lines at the same time, using the same approach [23]. Thus, there are no studies which address the complexity of MMPs/inhibitors system in a multi-factorial context. Moreover, there are no reports comparing expression profiles of these important modulators of the metastatic process, in both a cell line model system and in breast tumor tissue samples.

Here, we analyzed the expression levels of MMPs and their inhibitors, by qRT-PCR, in a panel of five human breast cancer cell lines displaying different degrees of invasiveness and metastatic potential and in 72 primary breast cancer and 30 adjacent non-tumor tissue specimens. The results demonstrate a significant and positive correlation between the levels of MMPs and their inhibitors both in the cell line models and in tumor tissue samples, suggesting that the expression of these molecules, at least at the transcriptional level, might be regulated by common factors and signaling pathways. Therefore, deregulation of only one of these components could be followed by alterations of several other members of the proteases/inhibitors families. Therefore, we suggest that a multi-factorial analysis is crucial to properly evaluate metastasis.

## Methods

### Cell Culture and RNA extraction

Five human breast cancer cell lines displaying different degrees of invasiveness and metastatic potential were used in this study (Table 1). 10^4^cell/cm^2 ^were seeded onto uncoated or Matrigel-coated plastic dishes and incubated for 3 or 5 days. Cells were lysed in 4 M guanidine thiocyanate; 25 mM sodium citrate (pH 7.0); 0.1 M β-mercaptoethanol) and the lysate was placed on top a Cesium chloride cushion [5.7 M cesium chloride, 25 mM sodium acetate (pH 5.0)]. Samples were ultracentrifuged (100,000 g, at 20°C for 18 h); the RNA pellet was solubilized in Milli-Q H_2_O and stored at -70°C. The RNA quality was evaluated by electrophoresis in agarose gel containing 20 mM guanidine thiocyanate.

**Table 1:** General characteristics of previously described human breast cancer cell lines

Cell Line	Histopathologycal type	Origin	ER	PR	Invasive potential	Metastatic potencial
MCF-7	Invasive ductal carcinoma	Metastasis (pleural effusion)	+	+	+	--
ZR-75-1	Invasive ductal carcinoma	Metastasis (ascite)	+	+	++	--
MDA-MB-435	Invasive ductal carcinoma	Metastasis (pleural effusion)	-	-	+++	++++
MDA-MB-231	Invasive ductal carcinoma	Metastasis (pleural effusion)	-	-	++++	+
Hs578T	Carcinosarcoma	Tumor primary	-	-	++++	++++

Table based on previously published data [39–41].

ER: status of estrogen receptor; PR: status of progesterone receptor (Negative (-), Positive (+)).

^a^Invasive potential analyzed by chemoinvasion assays through of reconstituted basal lamina (Matrigel) in transwell plates.

^b^Metastatic potential verified through of spontaneous metastasis assays in immune deficient mice

### Patients

The tumor and adjacent non-tumor breast tissue samples used in this study were provided by the Tumor Bank of the A.C. Camargo Cancer Hospital and Alfredo Abrão Cancer Hospital. The study protocol was approved by the Ethics Committee of these hospitals, certifying these studies were conducted in accordance with the guidelines of the 1975 Declaration of Helsinki (Process number 20 of June 24^th^, 2006). Informed consent was obtained from all patients before or after surgery. All of these tissue fragments were obtained during mastectomy by cold scalpel. The adjacent non-tumor tissue was removed at least 4 cm from the tumor border. All tissue samples were dissected by a pathologist, with the help of magnifying glasses, during surgery. The samples were harvested by the pathologist and immediately transferred to liquid nitrogen. These frozen samples were processed for RNA extraction, after informed consent was obtained from the patients. The methodology used for RNA extraction from tissue specimens was that previously described for RNA extraction from cell culture. The RNA quality was evaluated by electrophoresis in agarose gel containing 20 mM guanidine thiocyanate. The RNA samples were quantified and similar quantities were used for cDNA synthesis. Of these, 11 (15.3%) were coded as stage I, 16 (22.2%) as stage IIA, 20 (27.8%) as stage IIB, 9 (12.5%) as stage IIIA, 11 (15.3%) as stage IIIB and 5 (6.9%) as stage IV. Lymph nodes were evaluated in these tumors samples: 41 (56.9%) displayed compromised lymph nodes. The status of the molecular markers estrogen receptor (ER) and progesterone receptor (PR) was analyzed in 56 tumors, of which 41 tumors (73.2%) were positive for ER and 30 (53.6%) for PR. These and other clinical pathological data are shown in the Additional Data (see Additional file 1).

### Quantitative RT-PCR

For cDNA synthesis, 1 μg of total RNA was reverse-transcribed using oligo-dT, random primers and Superscript Amplification System (Invitrogen). Quantitative RT-PCR was carried out using SYBR Green PCR Master Mix (Applied Biosystems). Table 2 shows the primers used, with the optimal concentration (in range of 100 nM to 600 nM). Cycling conditions were 50°C for 2 min, 95°C for 10 min followed by 10 cycles of 95°C for 15 sec and 60°C for 30 sec. For quantification, the target genes were normalized to the internal standard GAPDH, HPRT, β-actin and β-tubulin genes. The amplification efficiency analyzed was calculated for each gene from the given slope in linear regression curve of Ct values versus log of cDNA concentration. The corresponding PCR efficiency (E) of one cycle in the exponential phase was calculated according to the equation: E = 10^[-1/slope]^. Relative expression levels were calculated according to the previously described Pfaffl model [25].

**Table 2:** Sequence of primers used

Gene	Primer sequence
MMP-2	F: AGCTCCCGGAAAAGATTGATG
	R: CAGGGTGCTGGCTGAGTAGAT
MMP-9	F: CCTGGAGACCTGAGAACCAATC
	R: GATTTCGACTCTCCACGCATCT
MMP-14	F: GCAGAAGTTTTACGGCTTGCA
	R: TCGAACATTGGCCTTGATCTC
TIMP-1	F: CCGCAGCGAGGAGTTTCTC
	R: GAGCTAAGCTCAGGCTGTTCCA
TIMP-2	F: CGACATTTATGGCAACCCTATCA
	R: GCCGTGTAGATAAACTCTATATCC
RECK	F: TGCAAGCAGGCATCTTCAAA
	R: ACCGAGCCCATTTCATTTCTG
GAPDH	F: ACCCACTCCTCCACCTTTGA
	R: CTGTTGCTGTAGCCAAATTCGT
b-tulin	F: TCAACACCTTCTTCAGTGAAACG
	R: AGTGCCAGTGCGAACTTCATC
b-actin	F: GGCACCCAGCACAATGAAG
	R: CCGATCCACACGGAGTACTTG
HPRT	F: GAACGTCTTGCTCGAGATGTGA
	R: TCCAGCAGGTCAGCAAAGAAT

### Zymography

Gelatin zymography was used to analyze MMP-2 and MMP-9 protease activities in human breast cancer cell lines (Table 1). 5 × 10^4^cell/cm^2 ^was seeded onto plastic or Matrigel-coated plastic dishes. After 48 h, cultures were washed and replenished with their respective serum-free ATCC-recommended culture medium plus 0.1% BSA (Sigma) for 72 h. The protein content of the serum-free conditioned medium (CM) was measured using the Bradford method. 25 μg of protein of each CM were mixed with SDS sample buffer without reducing agent and heated at 50°C for 10 sec. Samples were resolved in 12% polyacrylamide gels copolymerized with 0.1% gelatin (Bio-Rad). After electrophoresis, gels were washed in 2% (v/v) Triton X-100 for 15 min at 37°C to remove SDS, and then incubated in developing buffer [50 mM Tris-HCl (pH 7.4), 10 mM CaCl_2 _and 5 μM ZnCl_2_] for 16–20 h at 37°C. Subsequently, gels were fixed and stained with 30% methanol and 10% glacial acetic acid containing 0.5% Coomassie blue R250 (Sigma). The protease activity was visualized as clear bands within the stained gel.

### Statistical Analysis

All statistical analyses were performed with the GraphPad Prism 4.0 program. Results are presented as mean ± standard deviation. Statistical significance was determined using Student's *t *test for analysis of only two populations. One way ANOVA variance analysis and Tukey-Kramer test were used to calculate p-values for multiple comparisons. Pearson correlation test was used for correlation analysis. The Chi-square test was used in analysis of quantitative variable association. Differences were considered significant at p < 0.05.

## Results

### mRNA expression and activity of MMPs and their inhibitors in human breast cancer cell lines

**Figure 1:**
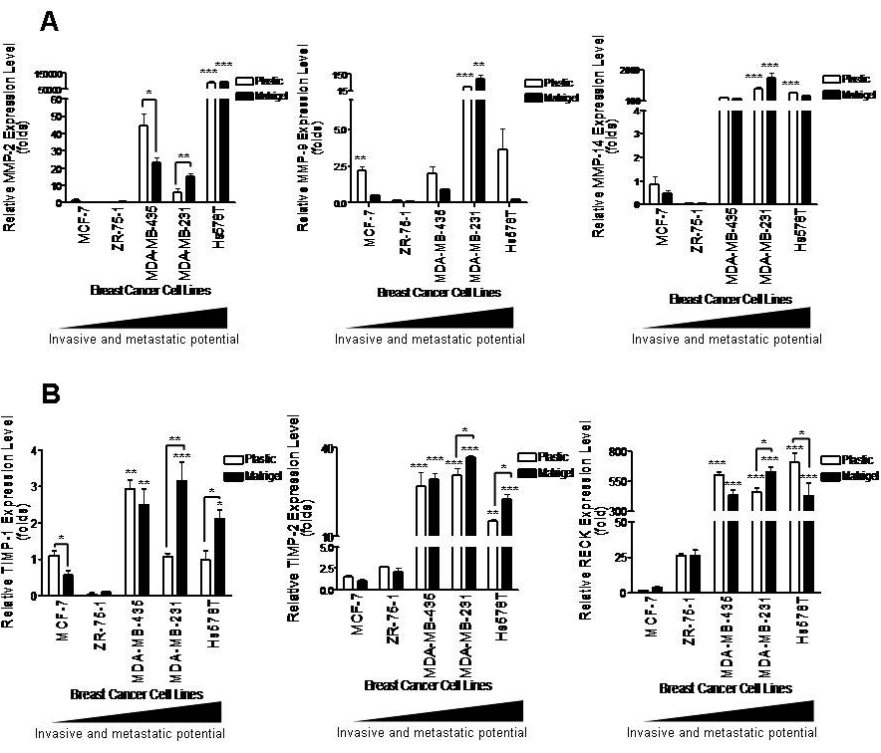
**Expression levels of MMPs (A) and their inhibitors (B) by qRT-PCR using total RNA from a panel of five human breast cancer cell lines, displaying increased degrees of invasiveness and metastatic potential, cultured on different substrates (plastic or Matrigel)**. GAPDH was used to normalize the values. Results are presented as MEANS ± SD of two independent experiments. P values were derived from One-way analysis of variance and Tukey-Kramer test. *, p < 0.05; **, p < 0.01 and ***p < 0.001, all versus control (MCF-7).

In order to determine whether MMPs and their inhibitors are responsible for the different invasive and metastatic capacities displayed by a panel of five human breast cancer cell lines (Table 1), we analyzed the expression levels of MMPs, TIMPs and RECK by qRT-PCR. The results (Fig. 1) demonstrated that the relative levels of MMP-2, MMP-9, MMP-14, TIMP-1, TIMP-2 and RECK were generally higher in highly invasive and metastatic cell lines (MDA-MB-435, MDA-MB-231 and Hs578T), when compared to less aggressive ones (MCF-7 and ZR-75-1). The levels of MMP-2 and MMP-14 were significantly elevated (p < 0.001) in the Hs578T cell line relative to those of less aggressive cell lines. The MDA-MB-231 cell line displayed significantly higher mRNA levels of MMP-9 and MMP-14 than MCF-7 (p < 0.001). In most cases, the MDA-MB-435 cell line showed a similar tendency, but it was not statistically significant. Moreover, all analyzed MMPs inhibitor (TIMP-1, TIMP-2 and RECK) are also over-expressed in MDA-MB-435, MDA-MB-231 and Hs578T breast cancer cells. The TIMP-1 gene expression was significantly higher in MDA-MB-435 than MCF-7 (p < 0.01). The others analyzed MMP inhibitors (TIMP-2 and RECK) displayed statically significant increased mRNA expression levels in all highly invasive and metastatic cell lines used in this study when compared with MCF-7 (p < 0.001, except to TIMP-2 expression in Hs578T whose statistical significance was p < 0.01.

**Figure 2:**
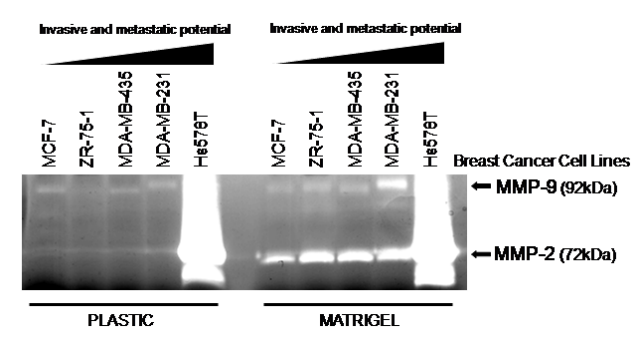
**Representative zymogram of gelatin zymography analysis to detect the activity of secreted MMP-2 and MMP-9 (three independent experiments) using conditioned medium from a panel of human breast cancer cell lines, displaying increased degrees of invasiveness and metastatic potential, cultured on different substrates (plastic or Matrigel)**.

Given that cell-ECM interaction regulates gene expression, we investigated the possible role of this interaction in the control of expression and activity of MMPs and their inhibitors. We cultured these human breast cancer cell lines on plastic or on artificial ECM (Matrigel) and then assessed MMPs and their inhibitors expression and activity. Among MMPs, only MMP-2 expression was significantly regulated by cell-ECM interaction, and only in MDA-MB-435 (p < 0.05) and MDA-MB-231 (p < 0.01) lines (Fig. 1A). However, the gelatinolytic activities of MMP-2 and MMP-9 are significantly higher in cells cultured on Matrigel than those seeded on uncoated plastic (Fig. 2). MDA-MB-231 and Hs578T cell lines displayed higher MMP activity than less invasive and metastatic breast cancer cell lines. The expression of all analyzed MMP inhibitors was modulated by cell-Matrigel contact in highly invasive and metastatic cell lines (MDA-MB-231 and Hs578T) (Fig. 1B). In almost all cases, the expression of these MMP inhibitors was not regulated by Matrigel. The results presented in Fig. 1 were obtained with cells cultured for five days on plastic or Matrigel. The same results were observed when cells were cultured for three days (data not shown). The relative mRNA expression data was normalized using two different housekeeping genes, namely: β-tubulin and GAPDH, and similar expression profiles were obtained.

### Correlation between mRNA expression of MMPs and their inhibitors in human breast cancer cell lines

We evaluated whether a correlation between mRNA expression levels of MMPs and their inhibitors exists in the cell model of tumor progression used in this study. The gene expression values displayed by these five human breast cancer cell lines cultured either on plastic or Matrigel for 3 or 5 days, obtained by qRT-PCR, were subjected to statistical analysis using Pearson correlation test. The results demonstrate a positive and significant correlation between the relative expression levels of all analyzed MMPs and MMP inhibitors. The expression of MMP-2 was an exception, since it significantly correlated only with MMP-14, TIMP-1 and RECK expression (Table 3).

**Table 3:** Correlation between MMPs and their inhibitors in human breast cancer cell lines

Pearson Correlation Test		R	p-value	n
MMP-2	MMP-9	-0.17	p = 0.1625	69
	MMP-14	0.47	*p < 0.0001*	72
	TIMP-1	0.25	*p = 0.0346*	72
	TIMP-2	0.10	p = 0.3979	69
	RECK	0.44	*p < 0.0001*	72

MMP-9	MMP-14	0.75	*p < 0.0001*	70
	TIMP-1	0.36	*p = 0.0022*	71
	TIMP-2	0.52	*p < 0.0001*	67
	RECK	0.32	*p = 0.0059*	71

MMP-14	TIMP-1	0.52	*p < 0.0001*	75
	TIMP-2	0.67	*p < 0.0001*	70
	RECK	0.67	*p < 0.0001*	74

TIMP-1	TIMP-2	0.68	*p < 0.0001*	71
	RECK	0.66	*p < 0.0001*	75

TIMP-2	RECK	0.80	*p < 0.0001*	70

Correlations between gene expressions data were calculated using Pearson Correlation test.

(n) Number of analyzed samples

(r) Pearson correlation coefficient

### mRNA expression levels of MMPs and their inhibitors in samples of primary breast tumors and adjacent non-tumor tissues

**Figure 3:**
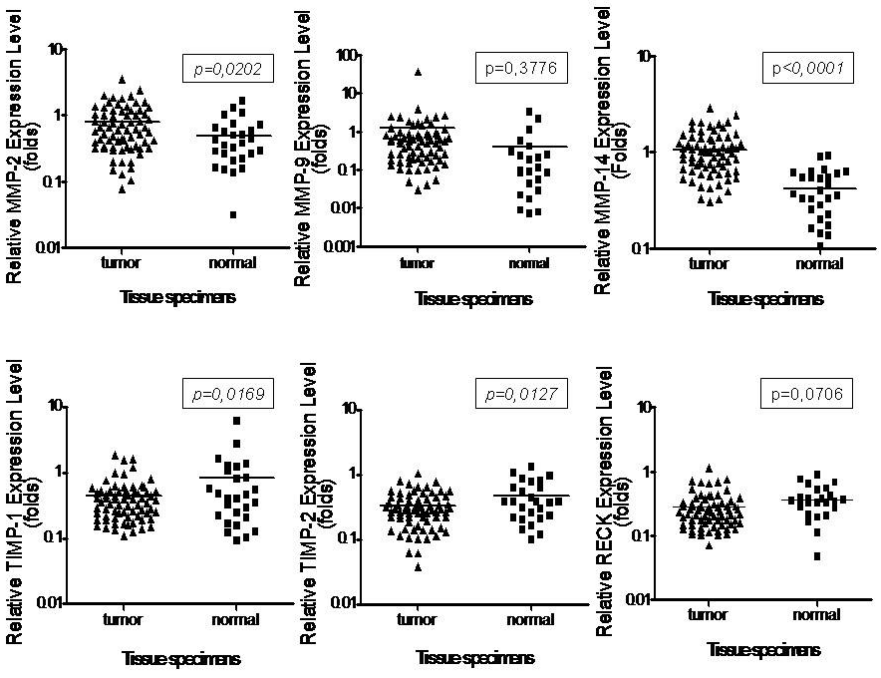
**Expression levels of MMPs and their inhibitors by qRT-PCR**. It was performed using total RNA from samples of primary breast tumors and adjacent normal border. β-actin was used to normalize the values. The statistical significance was determined using Student's *t *test.

In order to validate the data obtained from breast cancer cell lines (Fig. 1), 72 samples of invasive ductal breast carcinomas and 30 samples of adjacent non-tumor tissues were selected and analyzed by qRT-PCR. The results (Fig. 3) demonstrate that the relative expression levels of MMP-2 and MMP-14 of tumor samples were significantly higher than those of adjacent non-tumor tissue (p = 0.0202 and p < 0.0001, respectively). Furthermore, the samples derived from tumors showed a higher MMP-9 expression tendency than the adjacent non-tumor tissue specimens. TIMP-1 and TIMP-2 gene expression levels were significantly lower in tumors than in adjacent non-tumor tissue samples (p = 0.0169 and p = 0.0127, respectively). A similar trend was verified for RECK expression levels. These results were normalized using two different housekeeping genes (β-actin and HPRT), yielding similar expression profiles.

### Balance between relative gene expression levels of MMPs and their inhibitors in samples of primary breast tumors and adjacent non-tumor tissues

**Figure 4:**
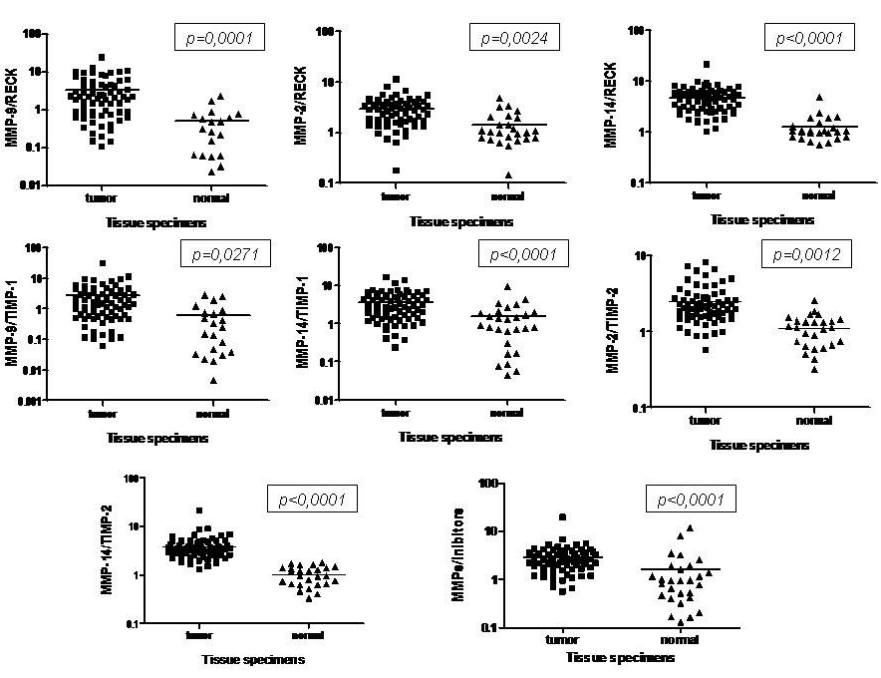
**Analysis of the proteinase/inhibitor balance at transcriptional levels by qRT-PCR using total RNA from samples of primary breast tumors and adjacent normal border**. β-actin was used to normalize the values. The statistical significance was determined using Student's *t *test.

The differences in the enzyme/inhibitor balance between tumor tissue specimens and adjacent non-tumor tissue samples were evaluated by calculating the MMPs/MMP inhibitors ratio values by using the mRNA expression levels data obtained by qRT-PCR. The results indicated that the ratios between MMP-2/RECK, MMP-9/RECK and MMP-14/RECK were significantly higher in the tumor than in adjacent non-tumor tissue samples (p = 0.0024, p = 0.0001 and p < 0.0001, respectively) (Figure 4). The same results were obtained for the ratios between MMP-9/TIMP-1, MMP-2/TIMP-2, MMP-14/TIMP-1 and MMP-14/TIMP-2 (p = 0.0271, p = 0.0012, p < 0.0001 and p < 0.0001, respectively). The same was obtained for the ratio between the sum of relative expression values of all MMPs (MMP-2, MMP-9 and MMP-14) and MMPs inhibitors (TIMP-1, TIMP-2 and RECK) (p < 0.0001)

### Correlation between MMPs, TIMPs and RECK expression and clinical-pathological variables in human breast carcinomas

The association of relative mRNA expression levels of MMPs, TIMPs and RECK with clinical-pathological data was statistically verified by Chi-Square test. MMP expression showed no significant association with the clinical data evaluated in this study (see Additional Data). The PR status presented a significant association with TIMP-1 gene expression (p = 0.020) (data not shown). Statistically significant associations were not observed between the relative expression of either TIMP-2 or RECK and the clinical variables analyzed.

The values of relative gene expression of MMPs and their inhibitors from breast tumor tissue samples were subjected to Pearson's test for correlation. The expression levels of TIMP-2 and RECK in tumoral samples showed a significant positive correlation with the gene expression of MMP-2 and MMP-14 (Table 4) (p < 0.0001 for all these correlations).

**Table 4:** Correlation between expression of MMPs and their inhibitors in samples of primary breast tumors

Pearson Correlation Test		r	p-value	N
MMP-2	MMP-9	0.06	p = 0.6523	67
	MMP-14	0.68	*p < 0.0001*	70
	TIMP-1	-1.3	p = 0.2968	70
	TIMP-2	0.65	*p < 0.0001*	70
	RECK	0.64	*p < 0.0001*	70

MMP-9	MMP-14	0.17	p = 0.1586	67
	TIMP-1	0.07	p = 0.6005	67
	TIMP-2	0.21	p = 0.0925	67
	RECK	0.04	p = 0.7245	67

MMP-14	TIMP-1	-1.7	p = 0.1474	70
	TIMP-2	0.61	*p < 0.0001*	70
	RECK	0.49	*p < 0.0001*	70

TIMP-1	TIMP-2	0.06	p = 0.6375	70
	RECK	-1.3	p = 0.2702	70

TIMP-2	RECK	0.42	*p = 0.0003*	70

Correlations between gene expressions data were calculated using Pearson Correlation test.

(n) Number of analyzed samples

(r) Pearson correlation coefficient

## Discussion

Development of metastasis is the most critical parameter for determination of survival in breast cancer patients. Patients diagnosed and treated before further extension of the tumor present higher cure rate [2-4]. The role of MMPs and their specific inhibitors in the metastatic process is well established. Nowadays, there are more than 20 MMPs identified in humans, and several MMP inhibitors, each one of these molecules being involved in different steps of tumor progression [20,21]. In spite of the complexity of MMPs/MMP inhibitors system, all previous studies which analyzed the status of these molecules during mammary tumor progression only focused on a small number of MMPs/MMP inhibitors in a limited number of models. Here, we proposed a more extensive approach by analyzing the mRNA expression levels of different MMPs (MMP-2, MMP-9 and MMP-14) and MMP inhibitors by qRT-PCR (TIMP-1, TIMP-2 and RECK) in several models (five human breast cancer cell lines, 72 primary breast tumors and 30 adjacent normal tissues).

The verified mRNA expression levels, obtained by qRT-PCR, suggested that breast cancer progression from pre-malignant stages to highly invasive and metastatic carcinomas could be accompanied by increased expression of MMPs and their inhibitors. The total RNA from a panel of five human breast cancer cell lines, displaying increased degrees of invasiveness and metastatic potential, were used to perform this quantitative RT-PCR assay. In the cell model used, the levels of all analyzed MMPs (MMP-2, MMP-9 and MMP-14) were, in almost all cases, higher in cell lines with elevated invasive and metastatic capacities (MDA-MB-435, MDA-MB-231 and Hs578T) when compared to less aggressive ones (MCF-7 and ZR-75-1). These results corroborate the data previously obtained by other groups, in which MMPs expression in breast cancer cell models positively correlates with a highly aggressive phenotype [26,27]. We found similar mRNA expression profiles for all analyzed MMP inhibitors (TIMP-1, TIMP-2 and RECK). At first sight, these results seem contradictory, since these molecules were described as MMP inhibitors. However, elevated mRNA expression levels of TIMP-1 and TIMP-2 have been previously detected in breast cancer cell lines with high invasive and metastatic potential [28]. Furthermore, several reports describe TIMPs as multifunctional molecules [8]. Thus, these molecules were both able to suppress and to promote tumor progression [8].

On the other hand, high expression levels of RECK, a membrane-associated MMP regulator protein, which is able to suppress tumor invasion and metastasis, were associated with better prognosis in several types of tumor, including breast carcinomas [18,19]. However, our results demonstrate that RECK mRNA expression level is increased during breast cancer progression. Thus, at first sight, these data seem to be at variance with the previous studies, suggesting that increased RECK levels could be associated not with better prognosis, but with late clinical stages. Nevertheless, other reports demonstrated that elevated levels of RECK were associated with better prognosis only for patients in late clinical stages [29,30]. Therefore, tumors that present overexpression of RECK could display a better clinical course, depending on the enzyme/inhibitor context. Thus, induction of RECK gene expression, observed during breast cancer progression, could be a cellular response to enhanced expression and activity of MMPs. In this context, the ECM proteolysis equilibrium could be maintained by re-establishment of the MMPs/inhibitors balance.

The role of cell-ECM elements interaction in regulation of expression and activity of these molecules was evaluated. The gelatin degradation ability of MMP-2 and MMP-9, evaluated by zymography assays, was significantly increased in cultures seeded onto Matrigel, when compared to those seeded onto uncoated plastic. Moreover, in highly aggressive cell lines, enhanced MMP activity was observed in less invasive and metastatic breast cancer cells, corroborating the data obtained in mRNA expression analysis. However, among the analyzed proteases, only MMP-2 expression was significantly modulated by cell-ECM elements interaction. These results suggest that increased proteolytic activity is independent of transcriptional activation, corroborating previously published data [31]. Our results demonstrate that the transcript levels of all studied MMP inhibitors are significantly modulated by the reconstituted basal lamina. This regulation of TIMP-1, TIMP-2 and RECK mRNA levels by cell-Matrigel contact was significant only in highly invasive and metastatic cell lines. Therefore, the intense ECM degradation is correlated with increased MMP inhibitor expression. The ECM supplies mechanical support to cells and structural integrity to tissues [6,32]. Furthermore, ECM performs an instructive role, since it is a reservoir of cytokines, and of growth and differentiation factors, which can be discharged and/or activated during proteolysis of ECM elements [33] by MMPs, contributing to the crosstalk between tumor cells and the microenvironment [34]. Therefore, the high mRNA expression levels of MMP inhibitors, found in highly aggressive cell lines, probably reflects a cellular reaction mechanism to intense ECM remodeling, as previously discussed. Thus, during ECM degradation, an important factor involved in induction of TIMPs and RECK can be discharged and/or activated. Our results corroborate this hypothesis, since we demonstrated that ECM is important only in modulation of gene expression of MMP inhibitors and, moreover, only in highly invasive and metastatic cell lines. Therefore, we suggest that this negative feedback mechanism of induction of protease inhibitors, during breast cancer progression, occurs for the re-establishment of the MMP/inhibitors balance and, consequently, for homeostasis of ECM proteolysis.

Cellular models are commonly used in Cell and Molecular Biology studies due to the limited source of tissue samples. However, enrichment for malignant cells and absence of stroma may compromise comparisons between cell lines and tumors [35]. Therefore, to discard possible cell culture artifacts, we also analyzed the expression of MMPs and their inhibitors in primary breast cancer tumor samples by qRT-PCR. The results obtained demonstrate, in a more general scenario, that the mRNA expression levels of MMPs are significantly higher in tumor samples than in adjacent non-tumor tissues. On the other hand, TIMP-1, TIMP-2 and RECK gene expression levels were significantly lower in tumors when compared with adjacent normal tissues. Furthermore, our results demonstrate a significant positive correlation between the gene expression levels of these proteases and their inhibitors in primary breast tumors from patients. Therefore, we suggest that increased expression of genes responsible for ECM degradation is coordinately accompanied by increased expression of their specific inhibitors and that the levels of MMPs, TIMPs and RECK transcripts may be regulated by common factors and signaling pathways.

Corroborating other studies in breast cancer [10,36], we found no significant association between the mRNA expression levels of the MMPs analyzed and clinical-pathological data [10,36]. These results suggest that the MMPs may supply independent prognostic information. However, there are conflicting results on the prognostic value of these proteases in this model. These differences can, at least in part, be due to the different methodologies used and to possible modulation of MMPs by adjuvant systemic treatment [37,38]. Furthermore, the TIMP-1, TIMP-2 and RECK transcript levels did not correlate with the clinical parameters from patients. The status of PR was an exception, which showed a significant correlation with TIMP-1 gene expression only. Likewise, we suggest that expression of the analyzed MMPs inhibitors may supply independent prognostic information.

## Conclusion

Taken together, the results obtained demonstrate that MMPs and their specific inhibitors are overexpressed in more aggressive breast cancer cell lines. However, it is likely that each one of these molecules plays distinct roles in acquisition of the invasive and metastatic capacity. This complexity of the MMPs-inhibitors system is intensified by a strong correlation found between the expression levels of these key regulators of the metastatic process. Thus, we suggest that the expression analysis of only one of these molecules or the independent quantification of these molecular markers does not provide sufficient information to elucidate the pathological proteolytic mechanisms associated with the tumor. Therefore, simultaneous analysis of the expression status of these molecules could be a better parameter to determine the most adequate therapy for each patient.

## Abbreviations

CM: conditioned medium; ECM: extracellular matrix; ER: estrogen receptor; MMP: matrix metalloproteinases; PR: progesterone receptor; qRT-PCR: quantitative reverse transcription polymerase chain reaction; RECK: reversion-inducing cysteine-rich protein with Kazal motifs; SD: standard deviation; TIMP: tissue inhibitors of matrix metalloproteinases

## Competing interests

The authors declare that they have no competing interests.

## Authors' contributions

RCSF was responsible for most of the experimental work and interpretation of some of the results. LRG was responsible for data discussion and interpretation, statistical analysis and manuscript preparation. JSN, FCS and IDCGS were responsible for collecting the tumor samples used in this study. MCS participated in the study design, data discussion and supervision of the study. All authors read and approved the final manuscript.

## Pre-publication history

The pre-publication history for this paper can be accessed here:

http://www.biomedcentral.com/1471-2407/9/20/prepub

## Supplementary Material

Additional file 1 **Clinic-pathological data. **The data provided represent the clinical pathological data from patients whose breast tumor or normal tissue samples were used in this study. Click here for file
