# Supplementary material for: Correlation between MMPs and their inhibitors in breast cancer tumor tissue specimens and in cell lines with different metastatic potential
Source: BMC Cancer. 2009 Jan 14;9:20. doi: 10.1186/1471-2407-9-20 (PMC2631003; doi:10.1186/1471-2407-9-20)
Supplement: Additional file 1 — Clinic-pathological data. The data provided represent the clinical pathological data from patients whose breast tumor or normal tissue samples were used in this study. [file 1471-2407-9-20-S1.doc]

Clinic-pathological data

| **Samples** | **Diagnostic date** | **TNM** | **T** | **M** | **Lymph node** | **Lymph node number** | **ER** | **PR** |
| --- | --- | --- | --- | --- | --- | --- | --- | --- |
| 36 | 06/21/01 | 2b | 2 | 0 | 1 | 2/29 | 0 | 1 |
| 38 | 02/14/02 | 3b | 4 | 0 | 0 | 0/29 | 1 | 0 |
| 39 | 03/14/02 | 2b | 2 | 0 | 1 | 10/21 | 1 | 1 |
| 40 | 04/02/2002 | 2b | 2 | 0 | 1 | 2/14 | 1 | 0 |
| 41 | 04/16/02 | 2b | 2 | 0 | 1 | 1/22 | 0 | 0 |
| 42 | 04/16/02 | 4 | 2 | 1 | 1 | 15/24 | 9 | 9 |
| 43 | 07/30/02 | 2b | 3 | 0 | 0 | 0/36 | 0 | 0 |
| 45 | 08/20/02 | 3a | 2 | 0 | 1 | 4/44 | 0 | 0 |
| 46 | 02/06/2001 | 2a | 2 | 0 | 0 | 0/20 | 1 | 0 |
| 51 | 04/26/01 | 2a | 2 | 0 | 0 | 1/23 | 1 | 1 |
| 52 | 03/22/01 | 2a | 2 | 0 | 0 | 0/23 | 0 | 0 |
| 54 | 10/24/00 | 2a | 1 | 0 | 0 | 1/18 | 1 | 1 |
| 55 | 02/20/01 | 2a | 2 | 0 | 0 | 0/14 | 1 | 1 |
| 57 | 09/03/2002 | 2b | 2 | 0 | 1 | 15/27 | 0 | 0 |
| 58 | 11/22/02 | 2a | 1 | 0 | 1 | 1/19 | 1 | 1 |
| 59 | 04/16/03 | 3a | 2 | 0 | 1 | 5/27 | 1 | 1 |
| 60 | 05/08/2003 | 1 | 1 | 0 | 0 | 0/15 | 0 | 0 |
| 61 | 05/03/2001 | 2a | 2 | 0 | 0 | 0/17 | 1 | 1 |
| 62 | 06/07/2001 | 2b | 2 | 0 | 1 | 3/31 | 1 | 1 |
| 63 | 06/26/01 | 2b | 2 | 0 | 1 | 125 | 1 | 1 |
| 64 | 09/06/2001 | 1 | 1 | 0 | 0 | 0/23 | 1 | 0 |
| 65 | 08/23/01 | 2b | 2 | 0 | 1 | 6/38 | 1 | 1 |
| 66 | 05/20/03 | 2a | 2 | 0 | 0 | 0/14 | 0 | 0 |
| 67 | 07/01/2003 | 3a | 3 | 0 | 1 | 13/33 | 1 | 1 |
| 68 | 07/17/03 | 3c | 3 | 0 | 1 | 15/51 | 0 | 0 |
| 69 | 07/17/03 | 3b | 4 | 0 | 1 | 8/16 | 0 | 0 |
| 70 | 08/05/2003 | 3a | 2 | 0 | 1 | 5/26 | 1 | 1 |
| 71 | 08/14/03 | 2b | 2 | 0 | 1 | 1/17 | 1 | 1 |
| 71 | 12/16/97 | 2b | 2 | 0 | 1 | 4/24 | 1 | 0 |
| 72 | 09/30/03 | 1 | 1 | 0 | 0 | 9 | 1 | 1 |
| 73 | 06/07/2001 | 2a | 1 | 0 | 1 | 1/17 | 1 | 1 |
| 158 | 02/17/98 | 2a | 2 | 0 | 0 | 0/24 | 1 | 1 |
|  |  |  |  |  |  |  |  |  |
| 181 | 03/05/98 | 2a | 1 | 0 | 1 | 3/19 | 1 | 0 |
| 921 | 05/13/99 | 2b | 2 | 0 | 1 | 2/21 | 0 | 0 |
| 1022 | 07/06/99 | 1 | 1 | 0 | 0 | 0/27 | 0 | 0 |
| 1263 | 11/09/99 | 2b | 2 | 0 | 1 | 3/30 | 1 | 0 |
| 1316 | 12/09/99 | 2b | 2 | 0 | 1 | 1/31 | 1 | 1 |
| 1403 | 02/17/00 | 3b | 4 | 0 | 0 | 0/10 | 0 | 0 |
| 1587 | 05/03/00 | 2a | 2 | 0 | 0 | 0/16 | 1 | 1 |
| 1658 | 06/13/00 | 3a | 2 | 0 | 1 | 13/43 | 1 | 1 |
| 12 | 12/09/03 | 2b | 2 | 0 | 1 | 14/16 | 9 | 9 |
| 15 | 12/15/03 | 4 | 4 | 1 | 1 | 11/13 | 9 | 9 |
| 23 | 01/27/04 | 4 | 2 | 1 | 1 | 05/05 | 9 | 9 |
| **Samples** | **Diagnostic date** | **TNM** | **T** | **M** | **Lymph node** | **Lymph node number** | **ER** | **PR** |
| 38 | 02/17/04 | 1 | 1 | 0 | 0 | 0/11 | 1 | 0 |
| 39 | 02/18/04 | 4 | 4 | 1 | 1 | 19/19 | 9 | 9 |
| 40 | 02/19/04 | 2b | 3 | 0 | 0 | 9 | 9 | 9 |
| 50 | 03/15/04 | 3b | 4 | 0 | 1 | 04/06 | 1 | 1 |
| 51 | 03/15/04 | 1 | 1 | 0 | 0 | 0/15 | 1 | 1 |
| 52 | 03/17/04 | 3b | 4 | 0 | 1 | 03/07 | 9 | 9 |
| 56 | 03/22/04 | 3a | 3 | 0 | 1 | 14/15 | 1 | 0 |
| 57 | 03/24/04 | 2b | 3 | 0 | 0 | 9 | 0 | 0 |
| 58 | 03/24/04 | 3b | 4 | 0 | 1 | 21/21 | 9 | 9 |
| 60 | 03/29/04 | 2b | 3 | 0 | 0 | 9 | 9 | 9 |
| 63 | 04/02/04 | 2a | 2 | 0 | 0 | 0/5 | 9 | 9 |
| 71 | 04/28/04 | 1 | 1 | 0 | 0 | 9 | 9 | 9 |
| 74 | 05/12/04 | 2b | 3 | 0 | 0 | 9 | 1 | 1 |
| 77 | 05/26/04 | 1 | 1 | 0 | 0 | 0/5 | 9 | 9 |
| 79 | 06/01/04 | 3a | 3 | 0 | 1 | 17/19 | 9 | 9 |
| 80 | 06/03/04 | 1 | 1 | 0 | 0 | 0/13 | 1 | 1 |
| 81 | 06/07/04 | 3a | 3 | 0 | 1 | 4/15 | 9 | 9 |
| 82 | 06/15/04 | 2a | 2 | 0 | 0 | 0/10 | 1 | 1 |
| 84 | 06/21/04 | 3b | 4 | 0 | 1 | 8/15 | 1 | 1 |
| 85 | 06/22/04 | 2a | 2 | 0 | 0 | 0/10 | 9 | 9 |
| 86 | 06/24/04 | 2b | 3 | 0 | 0 | 0/14 | 1 | 9 |
| 88 | 06/29/04 | 3b | 4 | 0 | 1 | 07/08 | 1 | 0 |
| 91 | 07/06/04 | 1 | 1 | 0 | 0 | 0/9 | 1 | 0 |
| 95 | 07/27/04 | 3b | 4 | 0 | 1 | 13/15 | 1 | 1 |
| 110 | 09/17/04 | 1 | 1 | 0 | 0 | 0/10 | 0 | 0 |
| 114 | 09/28/04 | 3a | 3 | 0 | 1 | 03/15 | 1 | 1 |
| 123 | 10/26/04 | 2a | 1 | 0 | 1 | 12/14 | 1 | 1 |
| 126 | 11/03/2004 | 4 | 4 | 1 | 1 | 9/14 | 9 | 9 |

**Samples:** tumor cadastre number at laboratory; **Diagnostic date:** tumor diagnostic date; **TNM:** staging of tumor by TNM system; **T**: tumor size; **M:** metastasis presence; **Lymph node:** presence of cancer cells found in any nodes; **Lymph node number:** Number of lymph nodes compromised; **ER:** Estrogen receptor state and **PR:** Progesterone receptor state. The number 9 in same quadrants represents that this parameter was not evaluated in this sample.
